# Supplementary material for: Cell culture–based production of defective interfering influenza A virus particles in perfusion mode using an alternating tangential flow filtration system
Source: Appl Microbiol Biotechnol. 2021 Sep 14;105(19):7251–64. doi: 10.1007/s00253-021-11561-y (PMC8437742; doi:10.1007/s00253-021-11561-y)
Supplement: Supplementary file 1 — Supplementary file1 (PDF 524 KB) [file 253_2021_11561_MOESM1_ESM.pdf]

Journal: “Applied Microbiology and Biotechnology”

Manuscript Title: “Cell culture-based production of defective interfering influenza A virus particles in perfusion mode using an alternating tangential flow filtration system”

Marc D. Hein[1], Anshika Chawla[2], Maurizio Cattaneo[3], Sascha Y. Kupke[2,*], Yvonne Genzel[2,*], Udo Reichl[1,2]

[1]Otto-von-Guericke-University Magdeburg, Chair of Bioprocess Engineering, Magdeburg, Germany

[2]Max Planck Institute for Dynamics of Complex Technical Systems, Bioprocess Engineering, Magdeburg, Germany

[3]Artemis Biosystems, Cambridge, Massachusetts, USA

*** Correspondence:**
Sascha Y. Kupke
E-mail: kupke@mpi-magdeburg.mpg.de
Phone: +49 391 6110-253
Fax: +49 391 6110-203

Yvonne Genzel
E-mail: genzel@mpi-magdeburg.mpg.de
Phone: +49 391 6110-257
Fax: +49 391 6110-203

Supplementary Material

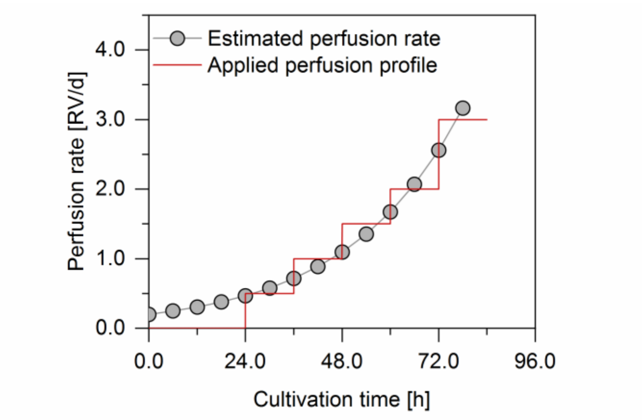

Fig. S1 Estimated and manually adjusted perfusion rate for cultivation of MDCK-PB2(sus) cells in a 1 L stirred tank bioreactor (HFM1). For the calculation of the required perfusion rate, the maximum specific growth rate (0.0354 1/h) and the glucose uptake rate (3.62E–10 mmol/cell/h) of a previous cultivation was used. Considering the metabolite uptake rates and the glucose (40 mmol/L) and glutamine (8 mmol/L) concentration in the Xeno™ medium, a cell specific perfusion rate (CSPR) of 200 pL/cell/d was calculated. Based on the expected cell growth and the CSPR the required perfusion rate was estimated. A stepwise perfusion profile was chosen accordingly.

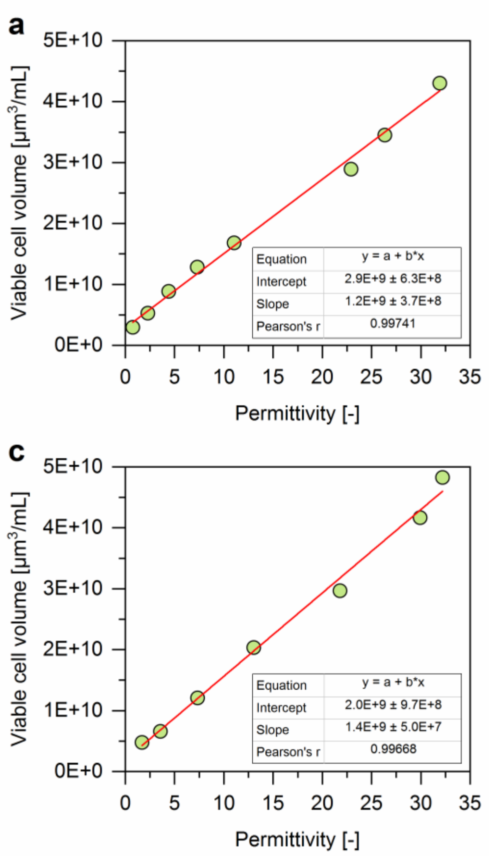

Fig. S2 Linear regression of the viable cell volume and permittivity measured for MDCK-PB2(sus) cells cultivated in a 1 L stirred tank bioreactor coupled to an alternating tangential flow filtration system (ATF 2). Perfusion cultivations were controlled using the capacitance probe for cell growth monitoring. As cell retention device a commonly used hollow fiber membrane (pore size of 0.2 µm; HFM2) or the virus harvest unit (pore size of ~10 µm; VHU1 and VHU2) was used. (a) HFM2, (b) VHU1, (c) VHU2.

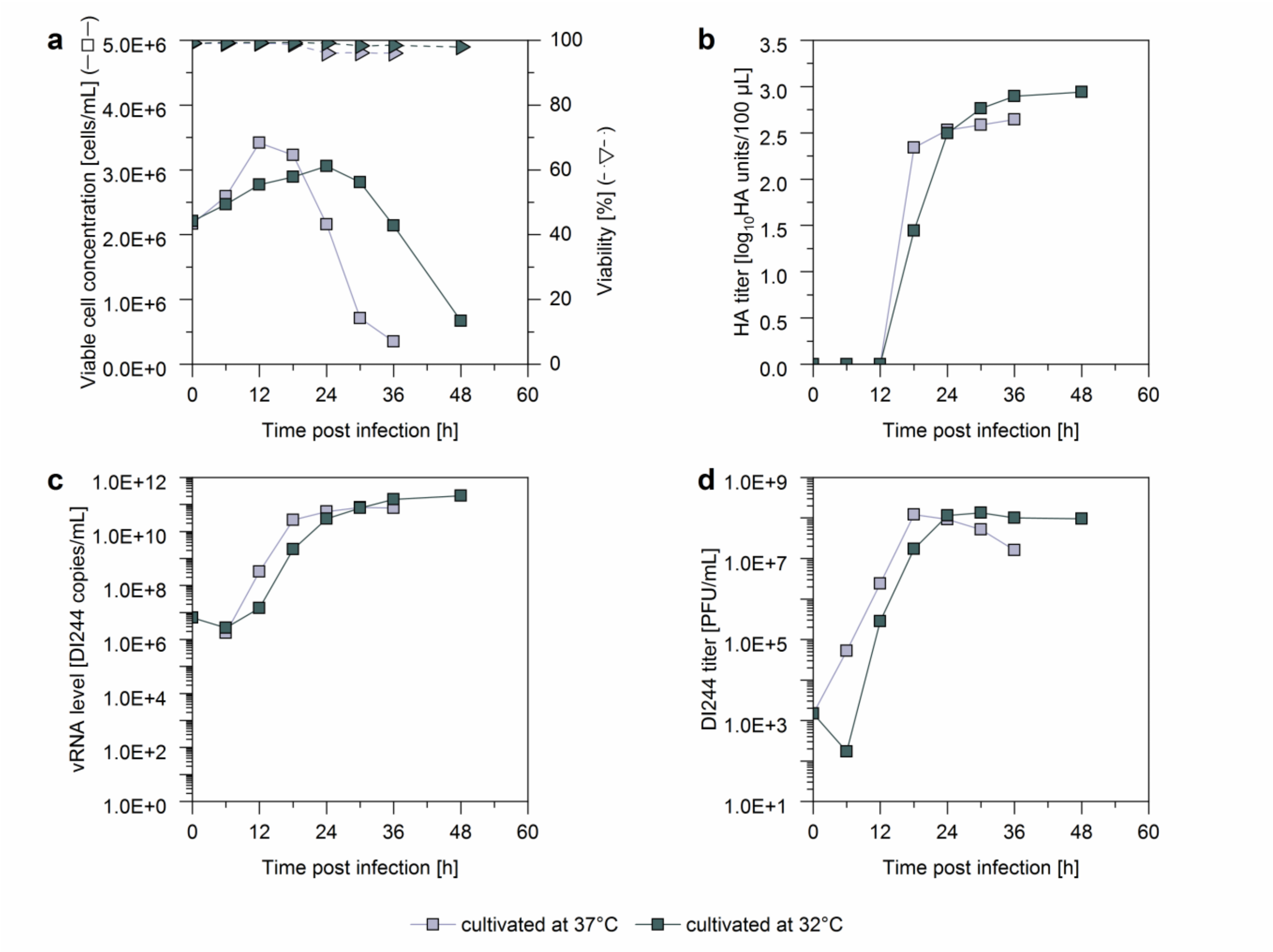

Fig. S3 Influenza A virus DIP production with MDCK-PB2(sus) cells cultivated in a shake flask (40 mL working volume) at different temperatures. At time of infection the viable cell concentration (VCC) was adjusted to 2.0E+6 cells/mL, the cells were infected with a pure DI244 seed virus at MODIP 1E–3. After infection, the cells were split and either cultivated at 37°C or 32°C. (a) VCC and viability, (b) HA titer, (c) vRNA level, and (d) DI244 titer.

Table S1 Overview of parameters for MDCK-PB2(sus) cells cultivated in a 1 L stirred tank bioreactor. For HFM1, HFM2, VHU1, and VHU2, the bioreactor was coupled to an alternating tangential flow filtration system (ATF2) to allow cultivation in perfusion mode. As cell retention device a hollow fiber membrane (pore size 0.2 µm; HFM1 and HFM2) or a virus harvest unit (pore size ~10 µm; VHU1 and VHU2) was used. For comparison the maximum specific growth rate and substrate uptake rates of the parental suspension MDCK cell line cultivated in a batch process are shown. Rates were determined for the exponential cell growth phase only.

Cultivation	Maximum specific growth rate [1/h]	Glucose uptake rate [mmol/cell/h]	Glutamine uptake rate [mmol/cell/h]
**Batch**	0.0354	3.62E-10	6.77E-11
**HFM1**	0.0363	2.37E-10	3.83E-11
**HFM2**	0.0299	1.61E-10	4.06E-11
**VHU1**	0.0328	1.99E-10	2.84E-11
**VHU2**	0.0355	2.30E-10	3.29E-11
